# The diagnostic value of DNA repair gene in breast cancer metastasis

**DOI:** 10.1038/s41598-020-76577-2

**Published:** 2020-11-12

**Authors:** Yongxin Yang, Xiabin Li, Liyue Hao, Deyong Jiang, Bin Wu, Tao He, Yan Tang

**Affiliations:** 1 Public Health Experimental Teaching Center, School of Public Health, Southwest Medical University, 1 Xianglin Road, Luzhou, 646000 Sichuan China; 2 Department of Pathology, The First Affiliated Hospital of Southwest Medical University, 25 Taiping Road, Luzhou, 646000 Sichuan China; 3 Sichuan Luzhou Center for Disease Control, 31 Datong Road, Luzhou, 646000 Sichuan China; 4 Department of Breast Surgery, First Affiliated Hospital of Southwest Medical University, 8 Kangcheng Road, Luzhou, 646000 China; 5 Institute of Cancer Medicine, School of Basic Medical Sciences, Southwest Medical University, 1 Xianglin Road, Luzhou, 646000 Sichuan China

**Keywords:** Cancer, Biomarkers

## Abstract

Breast cancer is the most common malignant tumor in China and even in the world. DNA repair genes can lead to tumor metastasis by affecting cancer cell resistance. Studies have preliminarily shown that DNA repair genes are related to breast cancer metastasis, but it is not clear whether they can be used as a prediction of the risk of breast cancer metastasis. Therefore, this study mainly discusses the predictive value of DNA repair genes in postoperative metastasis of breast cancer. The nested case–control method was used in patients with breast cancer metastasis after surgery (n = 103) and patients without metastasis after surgery (n = 103). The proteins and mRNA of DNA repair genes were detected by immunohistochemistry and Real-time PCR respectively. In protein expression, PARP1 (OR 1.147, 95% CI 1.067 ~ 1.233, *P* < 0.05), XRCC4 (OR 1.088, 95% CI 1.015 ~ 1.166, *P* < 0.05), XRCC1 (OR 1.114, 95% CI 1.021 ~ 1.215, *P* < 0.05), ERCC1 (OR 1.068, 95% CI 1.000 ~ 1.141, *P* < 0.10) were risk factors for postoperative metastasis of breast cancer. In addition, we used the ROC curve to study the optimal critical values of MSH2, MLH1, PARP1, XRCC1, XRCC4, 53BP1, ERCC1 and XPA combined with the Youden index, and the effects of MSH2, MLH1, PARP1, XRCC1, XRCC4, 53BP1, ERCC1 and XPA on breast cancer metastasis were verified again. Among them, the risk of metastasis in the PARP1 high expression group was 3.286 times that of the low expression group (OR 3.286, 95% CI 2.013 ~ 5.364, *P* < 0.05). The risk of metastasis in the XRCC4 high expression group was 1.779 times that of the low expression group (OR 1.779, 95% CI 1.071 ~ 2.954, *P* < 0.05). The risk of metastasis in patients with ERCC1 high expression group was 2.012 times that of the low expression group (OR 2.012, 95% CI 1.056 ~ 3.836, *P* < 0.05). So we can conclude that protein expression of PARP1 (cut-off value = 6, Se = 76.70%, Sp = 79.61%), XRCC4 (cut-off value = 6, Se = 78.64%0, Se = 79.61%), ERCC1 (cut-off value = 3, Se = 89.32%, Sp = 50.49%), suggesting that when the PARP1 score is higher than 6 or the XRCC4 score is higher than 6 or the ERCC1 score is higher than 3, the risk of metastasis will increases. Due to PARP1, XRCC4 and ERCC1 belong to a part of DNA repair gene system, and the three proteins are positively correlated by correlation analysis (*r*_PARP1-XRCC4_ = 0.343; *r*_PAPR1-ERCC1_ = 0.335; *r*_XRCC4-ERCC1_ = 0.388). The combined diagnosis of the PARR1, XRCC4 and ERCC1 have greater predictive value for the risk of metastasis of breast cancer (Se = 94.17%, Sp = 75.73%; OR 11.739, 95% CI 2.858 ~ 40.220, *P* < 0.05). The postoperative metastasis of breast cancer could be effectively predicted when the immunohistochemical scores met PARP1 (IHC score) > 6, XRCC4 (IHC score) > 6 and ERCC1 (IHC score) > 3. In addition, the combined diagnosis of PARP1, XRCC4 and ERCC1 has great predictive value for the risk of breast cancer metastasis.

## Introduction

Breast cancer is the most general malignancy in China and either world, and its mortality rate firstly in female malignancy^1^. In recent years, the survival rate of breast cancer has been prominently improved by comprehensive treatment such as surgery and chemotherapy^2^. Nevertheless, approximately one-third of breast cancer patients will present metastases^3^. Metastasis are bound up with the prognosis of breast cancer patients and it is also the soprattutto cause of death in breast cancer patients^4^. Studies have found that breast cancer patients’ postoperative metastasis are related to age, tumor pathological tissue type, clinical analysis, postoperative chemotherapy, and endocrine therapy^5^. At the same time, some people have also studied tolerance to treatment as one of the influencing factors. However, some tumor cells can pass activating self DNA repair mechanisms to resistance to DNA damage drugs^6–8^. So some studies have proposed that DNA repair genes have a relationship with the metastasis of breast cancer^9^.

More and more studies have found that tumor response to chemotherapy drugs is closely related to the regulation of the DNA repair system^10^. Four major DNA repair pathways are currently known: nucleotide excision repair (NER), base excision repair (BER), mismatch repair (MMR), and double strand break repair (DSBR). In cancer, we found that ERCC1, XPA, XRCC1, PARP1, MSH2, MLH1, 53BP1, XRCC4 are closely related to cancer metastasis^11–17^. The ERCC1 and XPA genes in the NER pathway have confirmed that ERCC1 is associated with metastasis in testicular germ cell tumors, and high expression of ERCC1 will lead to an increased risk of metastasis^18^. BER as one of the DNA repair mechanisms, PARP1 may be one of the major genes involved in tumor cell metastasis^19^. In vitro and in vivo studies have suggested that inhibition of PARP1 can reduce tumor cell repair function, thereby enhancing the therapeutic effect of radiotherapy and chemotherapy on tumors^20,21^. DSBR is the most common but most severe type of DNA damage in eukaryotic cells, and is mainly repaired in mammals through non-homologous end joining (NHEJ). Li et al. found that 53BP1 affects breast cancer patients’ sensitivity to 5-Fu, it will results poor prognosis^22^. MLH1, XRCC4, 53BP1, ERCC1 and XPA in breast cancer related studies, XRCC4 may be associated with breast cancer risk and the age at which breast cancer is diagnosed^23^, 53BP1 might be a crucial regulator of breast cancer migration and invasion^24^, women who can detect ERCC1 and XPA are at higher risk of breast cancer^25^, MLH1 and MSH2 loss may lead to advanced breast cancer^26^. XRCC1 overexpression can inhibit breast cancer cell proliferation and metastasis^27^. MSH2 mutation may be involved in the occurrence and development of early-onset breast cancer in the family of Lynch syndrome^28^. Among them, PARP1 inhibitors have entered the trial stage of clinical treatment of breast cancer^29^. But no further study of their metastasis with breast cancer.

DNA repair requires the role of multiple enzymes and genes. A single gene has a limited role in damage repair. Analyzing only an enzyme or gene is not enough to reflect the complexity of DNA repair. Due to ERCC1, XPA, XRCC1, PARP1, MSH2, MLH1, 53BP1, and XRCC4 are more studied in other cancer. But there are few studies in breast cancer metastasis. So in this study, nested case–control study was used to explore the expression levels of major molecules of the DNA repair system ERCC1, XPA, XRCC1, PARP1, MSH2, MLH1, 53BP1, and XRCC4 in patients with recurrent and metastatic breast cancer, in order to provide theoretical support for clinical treatment and prognosis.

## Methods

### Sample

The data come from the follow-up cohort of the Cancer Institute of Southwest Medical University. The cohort was collected and followed up in January 2013 at the Department of Breast Medicine, Southwest Medical University Hospital. Cancer patients have collected approximately 1360 cases. Metastasis cases and controls selected in this study were collected from this cohort. Patients with metastasis during the follow-up period were included in the metastasis case group. Metastasis definition: tumor cells leave the primary site of tumor formation and move to nearby or distal discontinuities and spread into macroscopic, clinically relevant masses the process^30,31^. At the same time, the control group(metastasis-free) was selected according to the 1:1 pairing principle in this cohort (n = 103, the matching condition was age ± 3 years, the operation time within the same month, and the treatment plan both are modified radical mastectomy). The control group (metastasis-free) was surviving patients in the cohort, and no metastasis occurred. Finally, 103 cases and 103 controls were included in January 2018. The average follow-up period was 31.25 months, the shortest follow-up period was 4 months, and the longest follow-up period was 59 months. The pathological data used in this study were from the Department of Pathology, Affiliated Hospital of Southwest Medical University. The data collected included clinical data, pathological data, and treatment options, as well as paraffin specimens from patients with breast cancer. After preliminary diagnosis of breast cancer patients in the affiliated hospital of Southwest Medical University, materials were obtained from the Department of Pathology. The paraffin blocks used in this study were sections by the co-author of this paper, Pathologist. Li Xiabin, and the samples were 100% tumor cells.

Ethical issues: (1) Patients with informed consent to participate. (2) The study plan has been reviewed by the Biomedical Ethics Committee of Southwest Medical University, and it is considered to meet the ethical requirements of clinical research, and the study plan is approved. Application acceptance Number: XNYD2018001.

### Detection of DNA repair genes ERCC1, XPA, XRCC1, PARP1, MSH2, MLH1, 53BP1 and XRCC4 in paraffin-embedded tissues of breast cancer patients by real-time PCR

Total RNA were extracted using the RNeasy FFPE Kit (QIAGEN, shanghai, China), according to manufacturer’s instructions. cDNA was reversely transcribed using the PrimeScript RT reagent Kit with gDNA Eraser (TaKaRa, Dalian, Liaoning, China).Gene expression was quantified by SYBR Premix Ex Tap II (TaKaRa, Dalian, Liaoning, China) and performed in a real-time thermal cycler qTOWER 2.0/2.2 (Analytik Jena, Germany) Relative gene expression was calculated using the 2^−ΔCT^ method and the results were normalized with β-actin as an internal control. The primer sequences are shown in Table 1.

**Table 1:** The primers used for PCR.

Gene	Primer sequences
53BP1	Sense primer 5′-CCAGACTCCACCAGACGAACA-3′ Anti-sense 5′-ACCACTTGGCTACAACACGGA-3′
ERCC1	Sense primer 5′-TATGAGCAGAAACCAGCGGAC-3′ Anti-sense 5′-GCTCGTGCAGGACATCAAACA-3′
MLH1	Sense primer 5′-TGAGGAAGGGAACCTGATTGG-3′ Anti-sense 5′-CCGGATGGAATAGAACATAGCG-3′
XRCC4	Sense primer 5′-TCTGTTCTGAAATGACTGCTGACC-3′ Anti-sense 5′-GGTGCAATATCAGTGACATCAAGAC-3′
MSH2	Sense primer 5′-GGAACTTCTACCTACGATGGATTTG-3′ Anti-sense 5′-TCAGTGGTGAGTGCTGTGACATG-3′
XRCC1	Sense primer 5′-TCGAGGACTATATGAGTGACCGG-3′ Anti-sense 5′-ACGAACGAATGCCAGGGAG-3′
PARP1	Sense primer 5′-CAGAAGCCGAAACTCTT-3′ Anti-sense 5′-GATGCCAAATCACCAGGT-3′
XPA	Sense primer 5′-TGTTTTGCCTCTGTTTTGGTT-3′ Anti-sense 5′-GTAATATGCGAAGAATGTGGG-3′
β-Actin	Sense primer 5′-CCACGAAACTACCTTCAACTCC-3′ Anti-sense 5′-GTGATCTCCTTCTGCATCCTGT-3′

### Immunohistochemical detection of DNA repair gene protein expression in paraffin-embedded tissues of breast cancer patients

Paraffin sections (3 μm) were dried, deparaffined, and rehydrated in graded alcohol to water. Heat-mediated antigen retrieval was performed using pressure cooker treatment for 10 min in EDTA buffer (pH 9.0). The slides were incubated for 120 min at 25 ℃ with primary mouse anti-human monoclonal antibodies to ERCC1, XPA, XRCC1, PARP1, MSH2, MLH1, 53BP1 and XRCC4 (Dako, DK). After washing, the sections were incubated with the second antibody (Envision, HRP rabbit/mouse, Dako, DK) for 30 min at 25 ℃. Negative controls were obtained by omitting the primary antibody. The slides were visualized by DAB.

Expression of 8 DNA repair protein was determined in the nucleus of tumor cells. Five high-power fields (200×) were randomly selected. The extent of the staining was categorized into five semi-quantitative classes based on the percentages of positive tumor cells: 0, < 5% positive cells; 1, 6–25% positive cells; 2, 26–50% positive cells; 3, 51–75% positive cells; and 4, > 75% positive cells. Staining intensity was scored as 0, negative; 1, weak; 2, moderate; and 3, intensive. Multiplication of the intensity and the percentage scores gave rise to the final staining score^32^.

### Statistical analysis

All data were analyzed using SPSS 22.0 statistical software and MedCalc software, and bilateral *P* values below 0.05 were considered statistically significant. Power test was (1 − β) = 0.9 used by statistics. The continuous variables in this study were all non-normal distributions, using the Wilcoxon signed-rank test in univariate analysis, and using the median (Interquartile Range) description. The correlation between DNA repair genes and breast cancer metastasis was analyzed by McNemar’s test, cox risk model and other statistical methods. Among DNA repair gene expression correlation this study adopts rank correlation method (Spearman rank correlation). The ROC curve was analyzed by MedCalc software.

### Ethics approval and consent to participate

Patients with informed consent to participate. The study plan has been reviewed by the Biomedical Ethics Committee of Southwest Medical University, and it is considered to meet the ethical requirements of clinical research, and the study plan is approved. Application acceptance Number: XNYD2018001. We confirming all the experiment protocol for involving humans was in accordance to guidelines of national in the manuscript.

### Consent for publication

All authors agree to submit the article for publication.

## Results

### The protein expression of DNA repair genes

**Figure 1:**
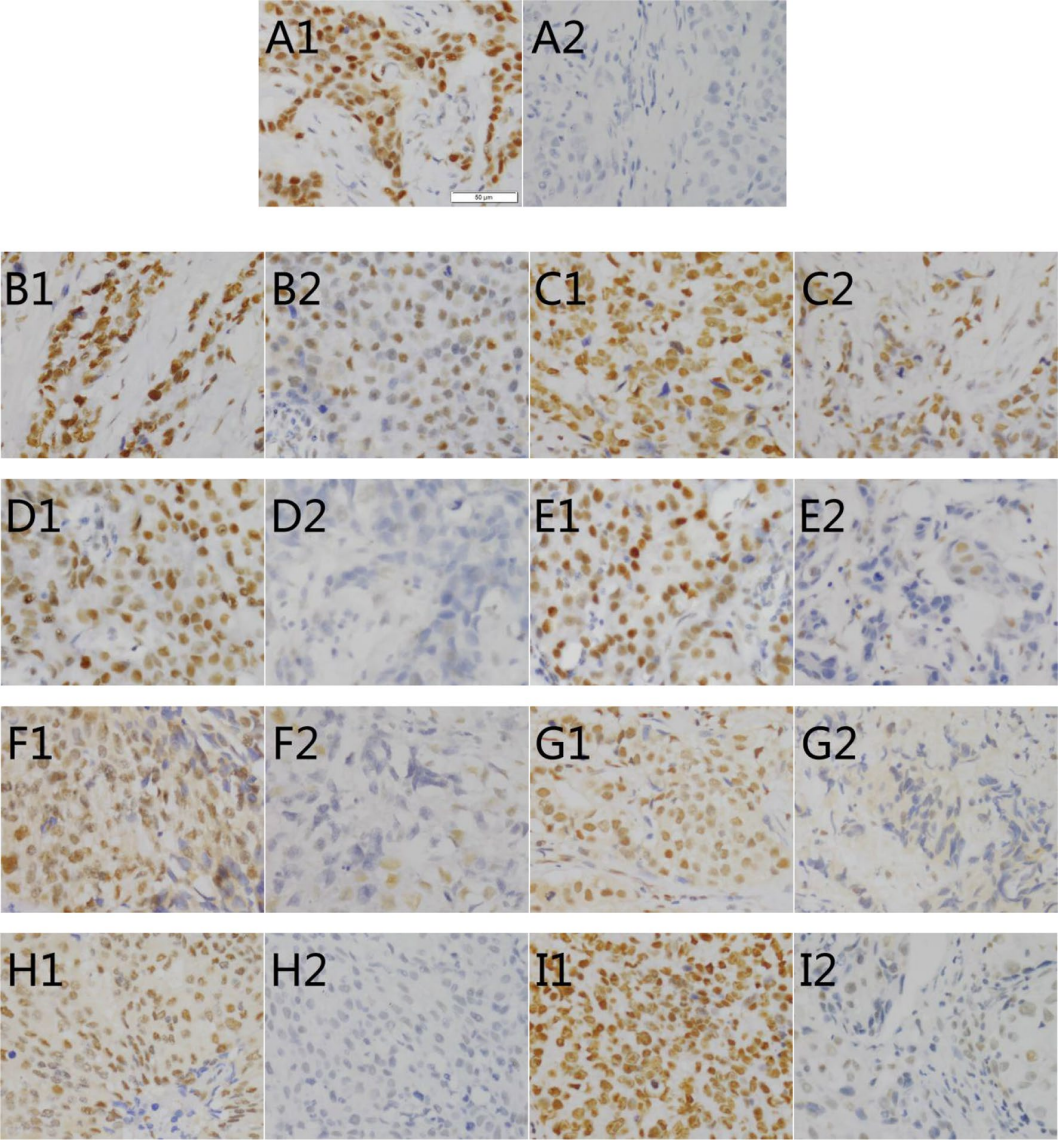
Strong expression of immunohistochemical positive controls compared to negative controls (**A**). Immunohistochemistry (IHC) detection of DNA repair genes MSH2 (**B**), MLH1 (**C**), PARP1 (**D**), XRCC1 (**E**), XRCC4 (**F**), 53BP1 (**G**), ERCC1 (**H**), XPA (**I**) in paraffin tissues of patients with metastasis breast cancer (1 for the metastasis group, 2 for the control group (metastasis-free group); original magnification × 400).

**Figure 2:**
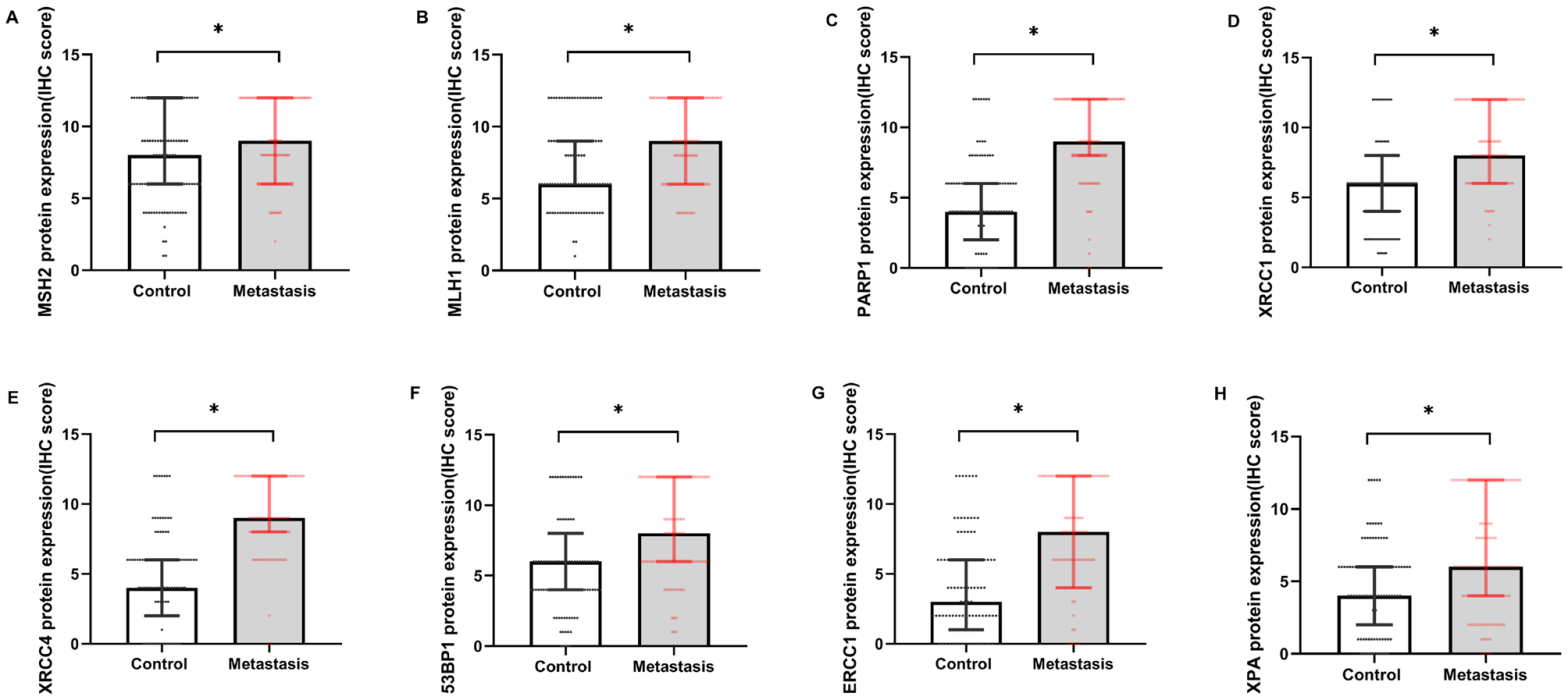
Shows the effect of breast cancer metastasis on the protein expression of DNA repair gene MSH2 (**A**), MLH1 (**B**), PARP1 (**C**), XRCC1 (**D**), XRCC4 (**E**), 53BP1 (**F**), ERCC1 (**G**), XPA (**H**) as shown in figure. Data are described as Median (Interquartile Range), N = 206. Statistical differences are expressed as: *P < 0.05.

Immunohistochemical staining results (Fig. 1) shows that: DNA repair gene protein positive expression mainly in the cytoplasm, repair gene is highly expressed in the metastasis group in the breast tissue. The MSH2, MLH1, PARP1, XRCC1, XRCC4, 53BP1, ERCC1, XPA of the metastasis group were higher than those of the control group (metastasis-free group) (*P* < 0.05), we can concluded that all of them are related to the prognosis of metastasis of breast cancer (Fig. 2).

### The mRNA expression of DNA repair genes

**Figure 3:**
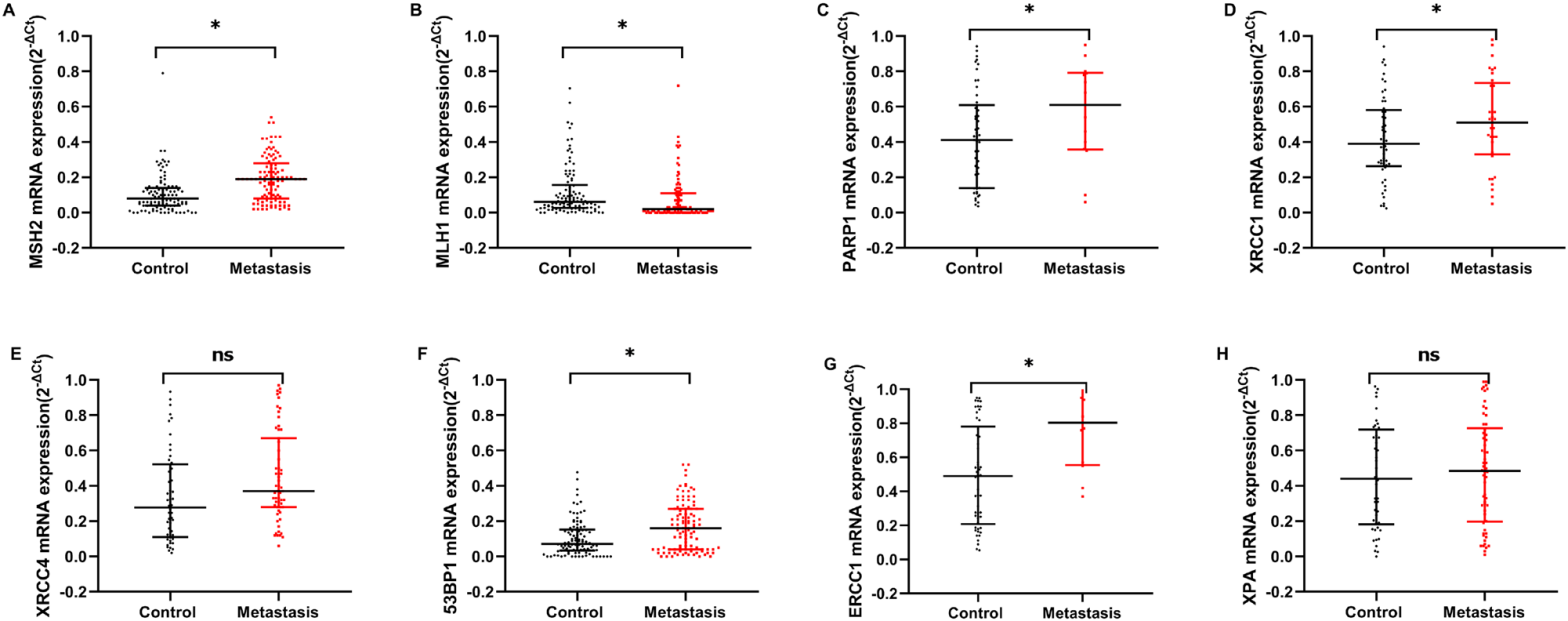
Shows the effect of breast cancer metastasis on the mRNA expression of DNA repair gene MSH2 (**A**), MLH1 (**B**), PARP1 (**C**), XRCC1 (**D**), XRCC4 (**E**), 53BP1 (**F**), ERCC1 (**G**), XPA (**H**). Data are described as Median (IQR), N = 206. Statistical differences are expressed as: *P < 0.05.

Figure 3 shows the comparison of the expression of DNA repair gene mRNA in breast cancer patients in the metastasis group and the control group (metastasis-free group). The mRNA expressions of MSH2, MLH1, PARP1, XRCC1, 53BP1, and ERCC1 in breast cancer metastasis group were higher than those in control group (metastasis-free group) (*P* < 0.05). There was no significant difference in XRCC4 and XPA between control group and metastasis group (*P* > 0.05).

### Clinicopathologic feature of breast cancer patients

The HER2, E-Cad, Ki67, Molecular subtypes and lymph node metastasis of the metastasis group was higher than that of the control group (metastasis-free group) (*P* < 0.10). The ER of the metastasis group was lower than that of the control group (metastasis-free group) (*P* < 0.05).There was no significant difference in Age, PR, P53, Pathological type, Tumor size and WHO Grade between the two groups (*P* > 0.10), as shown in Table 2.

**Table 2:** Clinicopathologic feature of breast cancer patients [n(%)].

Variable	Total n = 206	Control (non metastasis) n = 103	Metastasis n = 103	*P*-value
**Age**				
< 50	98	47 (45.6)	51 (49.5)	0.577^†^
≥ 50	108	56 (54.4)	52 (50.5)	
**ER**				
Negative	79	33 (32.0)	46 (44.7)	0.032*
Positive	127	70 (68.0)	57 (55.3)	
**PR**				
Negative	108	46 (44.7)	62 (60.2)	0.714*
Positive	98	57 (55.3)	41 (39.8)	
**HER2**				
−/+	138	68(66.0)	70(68.0)	0.001*
+++	68	35 (34.0)	33 (32.0)	
**E-Cad**				
Negative	24	14 (13.6)	10 (9.7)	< 0.001*
Positive	182	89 (86.4)	93 (90.3)	
**P53**				
Negative	107	58 (56.3)	49 (47.6)	0.757*
Positive	99	45 (43.7)	54 (52.4)	
**Ki67**				
< 20	54	31 (30.1)	23 (22.3)	< 0.001*
≥ 20	152	72 (69.9)	80 (77.7)	
**Molecular subtypes**				
Luminal A	36	22 (21.4)	14 (13.6)	0.064^†^
Luminal B	55	27 (26.2)	28 (27.2)	
Luminal HER2	45	25 (24.3)	20 (19.4)	
HER2-enriched	23	10 (9.7)	13 (12.6)	
Basal-like	47	19 (18.4)	28 (27.2)	
**Lymph node metastasis**				
0	71	49 (47.6)	22 (21.4)	0.000^†^
1 ~ 3	43	27 (26.2)	16 (15.5)	
4 ~ 9	38	12 (11.7)	26 (25.2)	
≥ 10	54	15 (14.6)	39 (37.9)	
**Pathological type**				
Carcinoma in situ	11	7 (6.8)	4 (3.9)	0.405^†^
Non-specific invasive carcinoma	193	95 (92.2)	98 (95.1)	
Invasive special type carcinoma	2	1 (1.0)	1 (1.0)	
**Tumor size**				
< 2 cm	60	33 (32.0)	27 (26.2)	0.819^†^
≥ 2 cm and ≤ 5 cm	120	55 (53.4)	65 (63.1)	
> 5 cm	26	15 (14.6)	11 (10.7)	
**WHO grade**				
I	9	8 (7.8)	1 (1.0)	0.465^†^
II	128	60 (58.3)	68 (66.0)	
III	69	35 (34.0)	34 (33.0)	

*P values were calculated by pairwise comparisons from McNemar’s test. ^†^P values were calculated by comparisons of groups from Wilcoxon signed-rank test.

### Cox regression analysis

To reduce confounding bias, at the protein expression level and the mRNA expression level, respectively, cox regression analysis was performed on variables related to prognosis in univariate analysis. The results showed that at the protein level, PARP1 (OR 1.147, 95% CI 1.067 ~ 1.233, *P* < 0.05), XRCC4 (OR 1.088, 95% CI 1.015 ~ 1.166, *P* < 0.05), XRCC1 (OR 1.114, 95% CI 1.021 ~ 1.215, *P* < 0.05), ERCC1 (OR 1.068, 95% CI 1.000 ~ 1.141, *P* < 0.10) and lymph node metastasis(≥ 10) were risk factors for postoperative metastasis of breast cancer. ER, HER2, E-Cad, Ki67, Molecular subtypes, MSH2, MLH1, 53BP1, XPA were not independent prognostic factors of postoperative breast cancer metastasis (*P* > 0.05).

The results of mRNA levels showed that the lymph node metastasis (4 ~ 9 or ≥ 10), MSH2 (OR 1.027, 95% CI 1.012 ~ 1.044, *P* < 0.05), PARP1 (OR 1.052, 95% CI 1.026 ~ 1.080, *P* < 0.05) were risk factors for postoperative metastasis of breast cancer. MLH1 (OR 0.066, 95% CI 0.009 ~ 0.0.484, *P* < 0.05), was protective factor for postoperative metastasis of breast cancer. ER, HER2, E-Cad, Ki67, Molecular subtypes, XRCC1, XRCC4, 53BP1, ERCC1 and XPA were not independent prognostic factors of postoperative breast cancer metastasis (*P* > 0.05). The variable assignment table is shown in Table 3. For details, see Tables 4 and 5.

**Table 3:** The variable assignment of cox model.

Variable	Variable assignment
Outcome	0 = control; 1 = metastasis
ER	0 = negative; 1 = positive
HER2	0 = negative; 1 = positive
E-Cad	0 = negative; 1 = positive
Ki67	1 = ‘< 20’; 2 = ‘ ≥ 20’
Molecular subtypes	1 = Luminal A; 2 = Luminal B; 3 = Luminal HER2; 4 = HER2-enriched; 5 = Basal-like
Lymph node metastasis	0 = ‘0’; 1 = ‘1 ~ 3’; 2 = ‘4 ~ 9’; 3 = ‘ ≥ 10’

**Table 4:** Cox regression of protein expression in metastasis of breast cancer.

Variable	*B*	SE	Wald	*P*-value	OR	OR 95% CI	
ER	− 0.551	0.544	1.026	0.311	0.576	0.198	1.674
HER2	− 0.615	0.727	0.714	0.398	0.541	0.130	2.250
E-Cad	0.158	0.384	0.170	0.680	1.171	0.552	2.487
Ki67	0.357	0.466	0.585	0.445	1.428	0.573	3.563
**Molecular subtypes**							
Luminal A	–	–	–	–	Reference	Reference	Reference
Luminal B	− 0.332	0.602	0.305	0.581	0.717	0.220	2.334
Luminal HER2	− 0.194	0.631	0.094	0.759	0.824	0.239	2.839
HER2-enriched	− 0.232	0.694	0.112	0.738	0.793	0.204	3.088
Basal-like	− 0.615	0.727	0.714	0.398	0.541	0.130	2.250
**Lymph node metastasis**							
0	–	–	–	–	Reference	Reference	Reference
1 ~ 3	− 0.066	0.344	0.037	0.847	0.936	0.477	1.836
4 ~ 9	0.419	0.315	1.765	0.184	1.521	0.819	2.822
≥ 10	0.786	0.332	5.599	0.018	2.195	1.144	4.208
MSH2	− 0.064	0.041	2.453	0.117	0.938	0.865	1.016
MLH1	− 0.069	0.045	2.341	0.126	0.934	0.855	1.020
PARP1	0.137	0.037	13.868	< 0.001	1.147	1.067	1.233
XRCC1	0.108	0.044	5.930	0.015	1.114	1.021	1.215
XRCC4	0.084	0.035	5.728	0.017	1.088	1.015	1.166
53BP1	− 0.020	0.034	0.352	0.553	0.980	0.918	1.047
ERCC1	0.066	0.034	3.805	0.051	1.068	1.000	1.141
XPA	0.040	0.030	1.775	0.183	1.041	0.981	1.103

**Table 5:** Cox regression of mRNA expression in metastasis of breast cancer.

Variable	*B*	SE	Wald	*P*-value	OR	OR 95% CI	
ER	− 0.194	0.217	0.799	0.372	0.823	0.538	1.261
HER2	− 0.338	0.228	2.204	0.138	0.713	0.456	1.114
E-Cad	− 0.163	0.363	0.203	0.653	0.849	0.417	1.729
Ki67	0.114	0.270	0.180	0.671	1.121	0.661	1.903
**Molecular subtypes**							
Luminal A	–	–	–	–	Reference	Reference	Reference
Luminal B	0.123	0.567	0.047	0.828	1.131	0.372	3.44
Luminal HER2	0.144	0.633	0.052	0.82	1.155	0.334	3.992
HER2-enriched	− 0.016	0.698	0.001	0.982	0.984	0.251	3.863
Basal-like	− 0.338	0.228	2.204	0.138	0.713	0.456	1.114
**Lymph node metastasis**							
0	–	–	–	–	Reference	Reference	Reference
1 ~ 3	0.149	0.335	0.198	0.656	1.161	0.602	2.238
4 ~ 9	0.702	0.307	5.208	0.022	2.017	1.104	3.685
≥ 10	1.116	0.280	15.886	< 0.001	3.053	1.763	5.286
MSH2	0.027	0.008	11.605	0.001	1.027	1.012	1.044
MLH1	− 2.723	1.019	7.146	0.008	0.066	0.009	0.484
PARP1	0.051	0.013	15.462	< 0.001	1.052	1.026	1.080
XRCC1	− 0.004	0.006	0.439	0.508	0.996	0.985	1.008
XRCC4	− 0.053	0.033	2.545	0.111	0.949	0.889	1.012
53BP1	− 0.383	0.891	0.185	0.667	0.682	0.119	3.905
ERCC1	0.033	0.031	1.106	0.293	1.033	0.972	1.099
XPA	− 0.090	0.080	1.247	0.264	0.914	0.781	1.070

### Diagnostic value of DNA repair genes

In the univariate study, we found that the protein expression of MSH2, MLH1, PARP1, XRCC1, XRCC4, 53BP1, ERCC1 and XPA were related to the metastasis of breast cancer (*P* < 0.05). However, the effect of multivariate analysis is not good since the IHC score is a continuous variable and there is no accurate cut-off value for diagnosis. In order to further understand the role of DNA repair genes in the prognosis of breast cancer metastasis. Therefore, we used the ROC curve to study the optimal critical values of MSH2, MLH1, PARP1, XRCC1, XRCC4, 53BP1, ERCC1 and XPA, as shown in Table 6.

**Table 6:** The best diagnostic value of protein expression in DNA repair genes.

Indicator	Cut-off value	Sensitivity (%)	Specificity (%)	Youden index	AUC	AUC (95% CI)	
MSH2	> 6	72.82	48.54	0.2136	0.629	0.559	0.695
MLH1	> 9	37.86	79.61	0.1748	0.620	0.550	0.686
PARP1	> 6	76.70	79.61	0.5631	0.833	0.775	0.881
XRCC1	> 6	67.96	74.76	0.4272	0.771	0.707	0.826
XRCC4	> 6	78.64	79.61	0.5825	0.834	0.776	0.882
53BP1	> 4	84.47	45.63	0.3010	0.688	0.620	0.750
ERCC1	> 3	89.32	50.49	0.3981	0.744	0.679	0.802
XPA	> 9	25.24	95.15	0.2039	0.634	0.565	0.700

MSH2, MLH1, PARP1, XRCC1, XRCC4, 53BP1, ERCC1 and XPA were divided into the high expression group and the low expression group according to the cut-off value. The variable assignment table after ROC prediction grouping is shown in Table 7. The effects of MSH2, MLH1, PARP1, XRCC1, XRCC4, 53BP1, ERCC1 and XPA on breast cancer metastasis were verified again by Cox Regression Analysis. Among them, the risk of metastasis in the PARP1 high expression group was 3.286 times that of the low expression group (OR 3.286, 95% CI 2.013 ~ 5.364, *P* < 0.05). The risk of metastasis in the XRCC4 high expression group was 1.779 times that of the low expression group (OR 1.779, 95% CI 1.071 ~ 2.954, *P* < 0.05). The risk of metastasis in patients with ERCC1 high expression group was 2.012 times that of the low expression group (OR 2.012, 95% CI 1.056 ~ 3.836, *P* < 0.05). The risk of metastasis in patients with lymph node metastasis (≥ 10) was 1.912 times that of lymph node metastasis (0) (OR 1.912, 95% CI 1.110 ~ 3.294, *P* < 0.05). As shown in Table 8.

**Table 7:** The variable assignment table of cox model after ROC prediction grouping.

Variable	Variable assignment
Outcome	0 = control; 1 = metastasis
ER	0 = negative; 1 = positive
HER2	0 = negative; 1 = positive
E-Cad	0 = negative; 1 = positive
Ki67	1 = ‘< 20’; 2 = ‘ ≥ 20’
Molecular subtypes	1 = Luminal A; 2 = Luminal B; 3 = Luminal HER2; 4 = HER2-enriched; 5 = Basal-like
Lymph node metastasis	0 = ‘0’; 1 = ‘1 ~ 3’; 2 = ‘4 ~ 9’; 3 = ‘≥ 10’
MSH2	1 = ‘≤ 6’; 2 = ‘> 6’
MLH1	1 = ‘≤ 9’; 2 = ‘> 9’
PARP1	1 = ‘≤ 6’; 2 = ‘> 6’
XRCC1	1 = ‘≤ 6’; 2 = ‘> 6’
XRCC4	1 = ‘≤ 6’; 2 = ‘> 6’
53BP1	1 = ‘≤ 4’; 2 = ‘> 4’
ERCC1	1 = ‘≤ 3’; 2 = ‘> 3’
XPA	1 = ‘≤ 9’; 2 = ‘> 9’

**Table 8:** Cox regression of protein high expression and low expression in postoperative metastasis of breast cancer.

Variable	*B*	SE	Wald	*P*-value	OR	OR 95% CI	
ER	− 0.441	0.207	4.553	0.033	0.643	0.429	0.965
HER2	− 0.261	0.660	0.156	0.693	0.771	0.211	2.812
E-Cad	0.144	0.373	0.149	0.700	1.155	0.556	2.396
Ki67	0.308	0.477	0.416	0.519	1.361	0.534	3.468
**Molecular subtypes**							
Luminal A	–	–	–	–	Reference	Reference	Reference
Luminal B	− 0.161	0.341	0.222	0.637	0.851	0.436	1.662
Luminal HER2	− 0.517	0.370	1.949	0.163	0.596	0.289	1.232
HER2-enriched	0.061	0.411	0.022	0.883	1.062	0.475	2.376
Basal-like	0.310	0.341	0.827	0.363	1.364	0.699	2.661
**Lymph node metastasis**							
0	–	–	–	–	Reference	Reference	Reference
1 ~ 3	0.061	0.330	0.034	0.853	1.063	0.557	2.030
4 ~ 9	0.471	0.298	2.500	0.114	1.601	0.893	2.871
≥ 10	0.648	0.278	5.451	0.020	1.912	1.110	3.294
MSH2	− 0.109	0.257	0.181	0.671	0.897	0.542	1.483
MLH1	− 0.174	0.242	0.518	0.472	0.840	0.523	1.349
PARP1	1.190	0.250	22.646	< 0.001	3.286	2.013	5.364
XRCC1	0.226	0.238	0.901	0.342	1.253	0.786	1.997
XRCC4	0.576	0.259	4.958	0.026	1.779	1.071	2.954
53BP1	0.085	0.29	0.085	0.770	1.088	0.616	1.922
ERCC1	0.699	0.329	4.514	0.034	2.012	1.056	3.836
XPA	0.346	0.248	1.958	0.162	1.414	0.870	2.297

**Figure 4:**
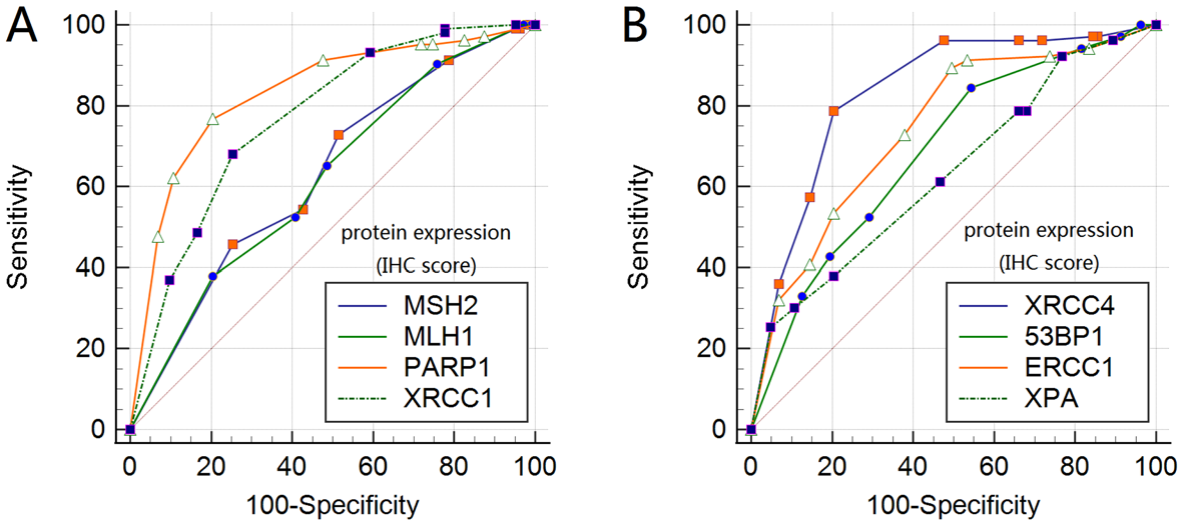
Diagnostic ROC curves of DNA repair genes protein expression. Diagnostic ROC curves of MSH2, MLH1, PARP1, XRCC1 protein expression (**A**); diagnostic ROC curves of XRCC4, 53BP1, ERCC1, XPA protein expression (**B**).

Combined with the sensitivity, specificity and Youden index, we can conclude that PARP1 (cut-off value = 6, Se = 76.70%, Sp = 79.61%), XRCC4 (cut-off value = 6, Se = 78.64%, Se = 79.61%), ERCC1 (cut-off value = 3, Se = 89.32%, Sp = 50.49%) have a good predictive effects, suggesting that when the PARP1 score is higher than 6 or the XRCC4 score is higher than 6 or the ERCC1 score is higher than 3, the risk of metastasis will increases. Diagnostic ROC curves of all genes as shown in Fig. 4.

### The correlate and joint diagnostic value on between PARP1, XRCC4 and ERCC1

**Figure 5:**
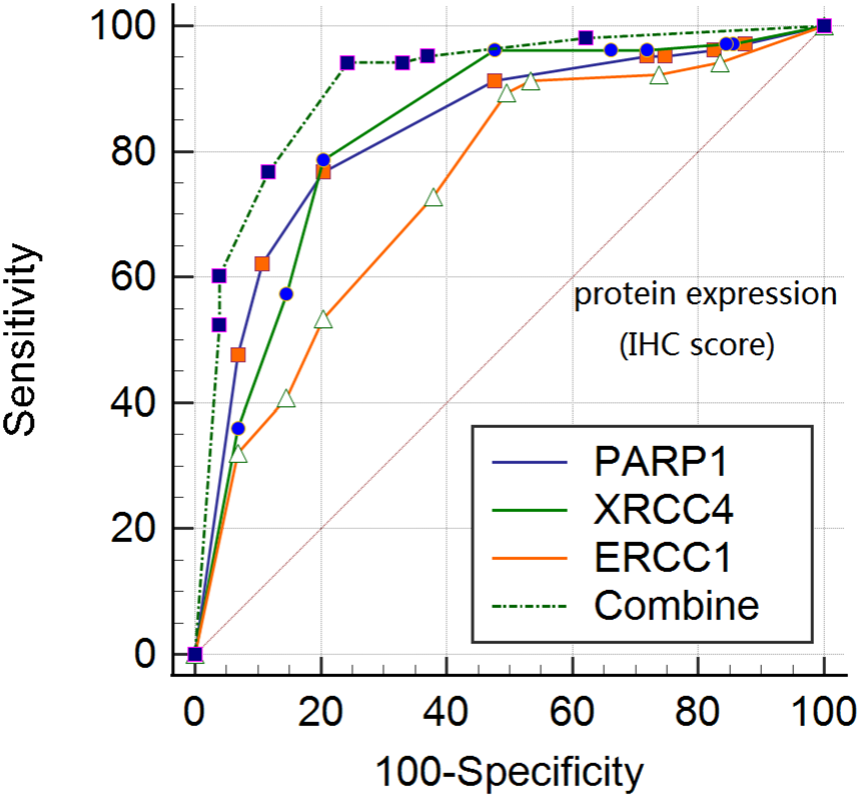
Comparison of PARP1, XRCC4 and ERCC1 ROC curves in diagnosis of breast cancer metastasis. Combine: PARP1 + XRCC4 + ERCC1.

Due to PARP1, XRCC4 and ERCC1 belong to a part of DNA repair gene system, and the three proteins are positively correlated by rank correlation analysis (*r*_PARP1-XRCC4_ = 0.343; *r*_PAPR1-ERCC1_ = 0.335; *r*_XRCC4-ERCC1_ = 0.388). See Table 9. And the correlation coefficient of mRNA expression in Table 10. These results indicate that there is an internal connection between these three proteins, and there is a certain synergy between them. So we combined PARP1, XRCC4 and ERCC1 to detect the prognosis of breast cancer. Joint diagnostic criteria: the high expression of a single indicator is judged as high, while the three indicators are simultaneously low and judged to be low (Se = 94.17%, Sp = 75.73%; AUC = 0.909, 95% CI 0.861 ~ 0.945). See Fig. 5 and Table 11. The correction effect of joint variables in multivariate, see Table 12.

**Table 9:** Correlation among the protein expressions of PARP1, XRCC4 and ERCC1 in breast cancer metastasis.

Variables	PARP1	XRCC4	ERCC1
PARP1	1	0.343*	0.335*
XRCC4	–	1	0.388*
ERCC1	–	–	1

Adopt rank correlation coefficient (Spearman) because protein data were not normal. *Refers to *P* < 0.05.

**Table 10:** The correlation coefficient of mRNA expression of MSH2, MLH1, PARP1 in breast cancer metastasis.

Variables	MSH2	MLH1	PARP1
MSH2	1	− 0.284*	0.401*
MLH1	–	1	− 0.029
PARP1	–	–	1

Adopt rank correlation coefficient (Spearman) because mRNA data were not normal. *Refers to *P* < 0.05.

**Table 11:** The Youden index and AUC of combine detection.

Joint indicator	Sensitivity (%)	Specificity (%)	Youden index	AUC	AUC (95% CI)	
PARP1 + XRCC4 + ERCC1	94.17	75.73	0.6990	0.909	0.861	0.945

**Table 12:** Cox regression of combined protein in postoperative metastasis of breast cancer.

Variable	*B*	SE	Wald	*P*-value	OR	OR 95% CI	
ER	− 0.322	0.496	0.420	0.517	0.725	0.274	1.917
HER2	− 0.492	0.704	0.489	0.484	0.611	0.154	2.429
E-Cad	− 0.145	0.357	0.166	0.684	0.865	0.430	1.739
Ki67	0.251	0.444	0.320	0.572	1.286	0.538	3.072
**Molecular subtypes**							
Luminal A	–	–	–	–	Reference	Reference	Reference
Luminal B	0.044	0.574	0.006	0.940	1.044	0.339	3.219
Luminal HER2	0.169	0.611	0.077	0.782	1.185	0.358	3.921
HER2-enriched	− 0.136	0.668	0.041	0.839	0.873	0.236	3.236
Basal-like	− 0.492	0.704	0.489	0.484	0.611	0.154	2.429
**Lymph node metastasis**							
0	–	–	–	–	Reference	Reference	Reference
1 ~ 3	− 0.097	0.332	0.085	0.771	0.908	0.474	1.740
4 ~ 9	0.693	0.305	5.175	0.023	2.000	1.101	3.632
≥ 10	0.974	0.294	10.967	0.001	2.648	1.488	4.713
PARP1 + XRCC4 + ERCC1	2.463	0.721	11.674	0.001	11.739	2.858	48.220

## Discussion

Chemotherapy is one of the most important treatments for breast cancer after operation. At present, the survival rate of patients has been effectively improved by referring to ER, PR, HER-2, Ki67, TNBC and other indicators. However, the study found that there are still about 30% metastasis rates^33^. It shows that the formulation of treatment plans based on the above pathological indicators may be incomplete, and there are other indicators for guiding treatment that can be excavated. Therefore, it is still necessary to improve the survival rate of patients when formulating treatment plans. However, the drug resistance of cancer cells is very common, which is the main reason for the failure of advanced breast cancer treatment and poor prognosis. Therefore, it is particularly important to solve the problem of breast cancer cell drug resistance, and the drug resistance of cancer cells is closely related to DNA repair genes. Today, more and more studies have found that tumor metastasis is closely related to the DNA repair regulatory system related to drug resistance^34–36^.

Many DNA repair genes such as MSH2, MLH1, PARP1, XRCC1, XRCC4, 53BP1, ERCC1, XPA have been found to be associated with the prognosis of breast cancer. PARP1, XRCC4, ERCC1 is also found to be an independent factor for postoperative metastasis of breast cancer. PARP1 promotes the expression of HIF-1α by activating nuclear factor-κB (NF-κB) and promotes the polarization of macrophages M2, leading to the up-regulation of tumor-related macrophages (TAMs), such as tumor necrosis factor-α (TNFα) and IL-6, thus promoting the proliferation, invasion and metastasis of tumor cells, promoting the formation of tumor microvessels and microlymphatics^37^. The up-regulation of NF-κB pathway expression and activation of cellular inflammatory response have also been reported to lead to PARP inhibitor resistance^38^. Tumor necrosis factor-α (TNFα) is closely related to the occurrence of cancer. The secretion of TNF-α cytokines in tumor microenvironment can accelerate the growth and spread of cancer cells. At the same time, it can make cancer cells bypass the impact of the immune system, promote the EMT process of cells, and cause distant metastasis of cancer^39^. XRCC4 is an important enhancer in promoting repair pathway triggered by DNA double-strand break (DSB). In the context of radiation therapy, active XRCC4 could reduce DSB-mediated apoptotic effect on cancer cells. Hence, developing XRCC4 inhibitors could possibly enhance radiotherapy outcomes^40^. And ERCC1 proteins can form heterodimers with DNA repair enzyme deficiency complementary gene (XPF) and perform functions by splicing at the 5′end of the damaged DNA single strand. Overexpression of ERCC1 proteins can lead to rapid repair of damaged DNA stagnating in G2/M, leading to resistance to cisplatin chemotherapeutics^41^.

The mRNA period of Real time-PCR detection is very short, generally only 30 min, and involves the problem of post-metastatic translation and time point, so there is mRNA expression, but not necessarily transcribed into protein, mRNA no expression may be in. Therefore, mRNA expression can not represent the final protein expression level, so in ROC curve analysis, this study uses IHC score to analyze. However, the direct use of IHC score to analyze the metastasis of breast cancer after surgery is of little significance. The scores of IHC scores are mostly 0 ~ 4, 6, 8, 9, 12, the scores are not completely continuous, the results are difficult to explain, and the OR has no clinical significance. In order to further understand the role of PARP1, XRCC4 and ERCC1 in predicting the prognosis, metastasis of breast cancer, we also studied the best cut-off value of PARP1, XRCC4 and ERCC1. The IHC scores of PARP1, XRCC4 and ERCC1 were higher than that of 6, 6 and 3 breast cancer metastasis, respectively. The sensitivity of PARP1, XRCC4 and ERCC1 single detection is between 67.96 ~ 89.32%, the specificity is between 50.49 ~ 79.61%, the Youden index is between 0.3981 ~ 0.5825, the sensitivity were reach the standard, but the specificity and Youden index were low. It indicates that the diagnostic value of individual tumor markers in the prognosis of breast cancer needs to be further improved. Due to PARP1, XRCC4 and ERCC1 belong to a part of DNA repair gene system, and the three proteins are positively correlated by correlation analysis. These results suggest that there is an internal link among the three proteins and there is a certain synergy among them. So we combined protein expression (IHC score) of PARP1, XRCC4 and ERCC1 to detect the prognosis of breast cancer. Joint diagnostic criteria: the high expression of a single indicator is judged as high, while the three indicators are simultaneously low and judged to be low. The results showed that after using the joint test, the specificity of diagnosis increased from 50.49 to 94.17%. The Youden index increased from 0.3981 to 0.6990. Sensitivity only decreased from 89.32 to 75.73%. And in the cox regression of breast cancer prognosis, the odds ratio of the combined indicators is as high as 11.739. It can be seen that the combined detection of three DNA repair proteins has higher clinical diagnostic value than the single determination. While both PARP1, XRCC4 and ERCC1 are related to tumor resistance and metastasis, the specific biological mechanism and the existence of a common mechanism of action between the three are unclear and need further study.

## Conclusions

The postoperative metastasis of breast cancer could be effectively predicted when the immunohistochemical scores met PARP1 (IHC score) > 6, XRCC4 (IHC score) > 6 and ERCC1 (IHC score) > 3. In addition, the combined diagnosis of PARP1, XRCC4 and ERCC1 has great predictive value for the risk of breast cancer metastasis. However, the mechanism of the effect of PARP1, XRCC4 and ERCC1 on the metastasis of breast cancer remains unclear, which needs further study (Fig. S1A1).

## Limitation and advantage of the study

This study is a prospective nested case–control study with complete data. Cases and controls in the study come from the same cohort, thus reducing the selection bias and comparability of effect estimation. Exposure data in the study were collected before disease diagnosis. If the results show that exposure is associated with disease, the association is consistent with the chronological order of causality inference, with less or avoidable recall, stronger causal inference, and higher statistical efficiency and test efficiency in nested case–control studies than in case–control studies, and disease frequency can be calculated. Save a lot of manpower, material and financial resources than the cohort study.

This study has only preliminarily explored the predictive value of DNA repair genes in postoperative metastasis of breast cancer, and has not further studied the regulatory mechanism of DNA repair genes in breast cancer metastasis and the screening of drug targets. Our group plans to carry out the next in-depth study.

## Supplementary information

Supplementary Information.

## Data Availability

The data and materials of this study are available from the corresponding authors for reasonable requests.

## Abbreviations

ERCC1: DNA excision repair cross complementing 1
XPA: Xeroderma pigmentosum group A
XRCC1: X-ray repair cross-complementing 1
PARP1: Poly ADP-ribose polymerase-1
MSH2: MutS homolog 2
MLH1: MutL homolog 1
53BP1: P53 binding protein
XRCC4: X-ray repair cross-complementing 4
ER: Estrogen receptor
PR: Progesterone receptor
HER2: Humanepidermalgrowthfactorreceptor-2
TNBC: Triple negative breast cancer
TAMs: Tumor-related macrophages
TNF-α: Tumor necrosis factor-α
DNA-PK: DNA-dependent protein kinase complex
EMT: Epithelial–mesenchymal transition
NF-κB: Nuclear factor-kappa B
5-Fu: 5-Fluorouracil
