# Supplementary material for: The diagnostic value of DNA repair gene in breast cancer metastasis
Source: Sci Rep. 2020 Nov 12;10:19626. doi: 10.1038/s41598-020-76577-2 (PMC7661505; doi:10.1038/s41598-020-76577-2)
Supplement: Supplementary file 1 — Supplementary Information. [file 41598_2020_76577_MOESM1_ESM.docx]

**Supplementary information**

**The diagnostic value of DNA repair gene in breast cancer metastasis**

Yongxin Yang^1,a^, Xiabin Li^2,a^, Liyue Hao^1^, Deyong Jiang^3^, Bin Wu^4^, Tao He^5*^, Yan Tang^1,5*^

**Author affiliations:**

1.Public Health Experimental Teaching Center, School of Public Health, Southwest Medical University, 1 Xianglin Road, Luzhou, Sichuan, 646000, China.

2.Department of Pathology, the First Affiliated Hospital of Southwest Medical University, 25 Taiping Road, Luzhou, 646000 Sichuan, China.

3.Sichuan Luzhou Center for Disease Control, 31 Datong Road, Luzhou, 646000 Sichuan, China.

4.Department of Breast Surgery, First Affiliated Hospital of Southwest Medical University, 8 Kangcheng Road, Luzhou, 646000, China.

5.Institute of Cancer Medicine, School of Basic Medical Sciences, Southwest Medical University, 1 Xianglin Road, Luzhou, 646000 Sichuan, China.

***Correspondence:**

Yan Tang (tangyan200310@163.com)

Public Health Experimental Teaching Center, School of Public Health, Southwest Medical University

1 Xianglin Road, Luzhou, Sichuan, 646000, China.

Phone: +86-180-0821-3621

**Running headline**: Immunohistochemical staining

Supplementary information: 1 figure


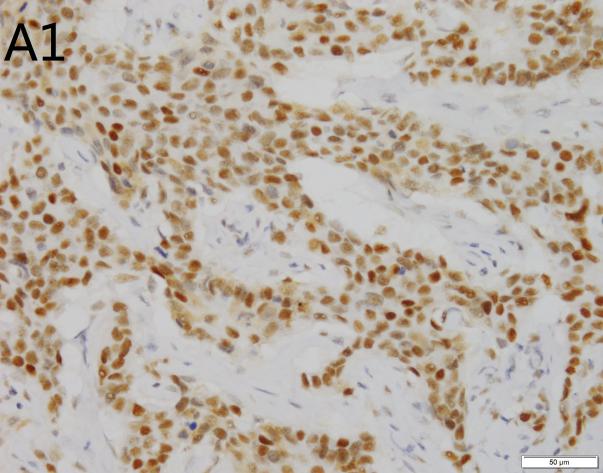

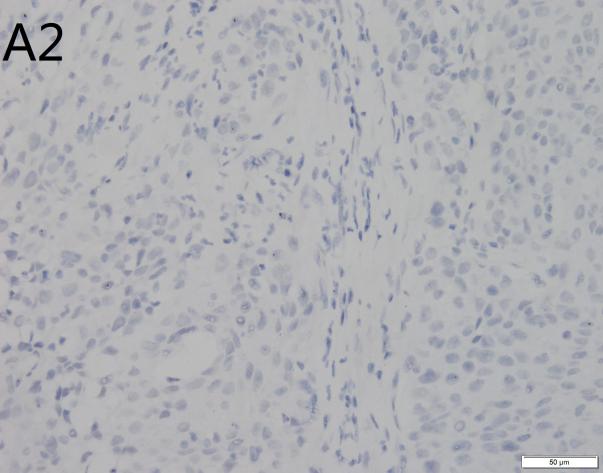

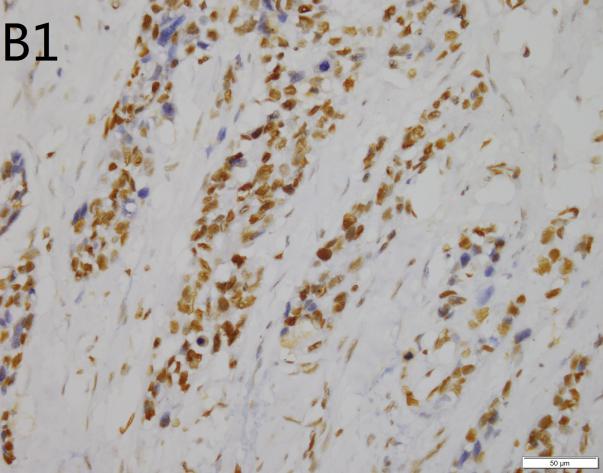

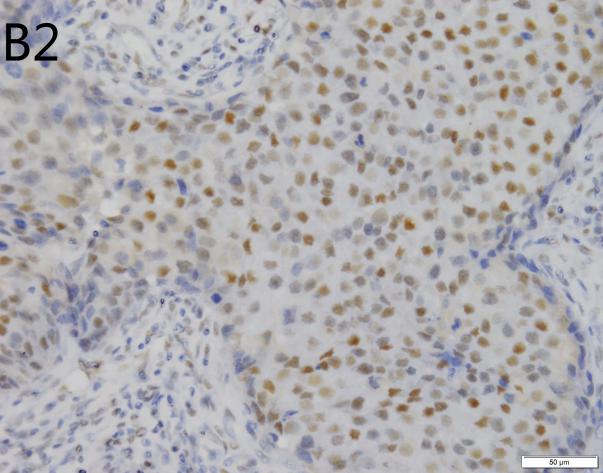

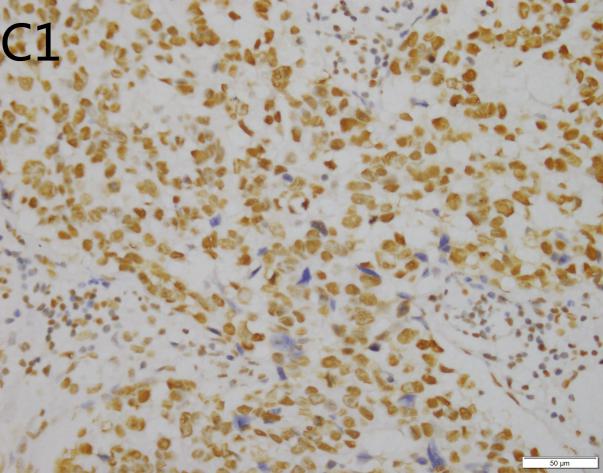

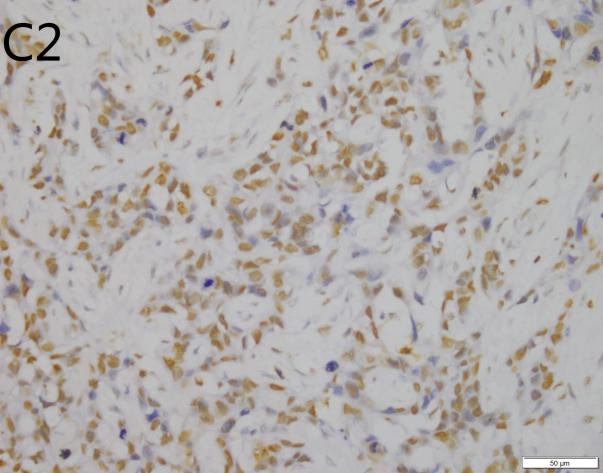

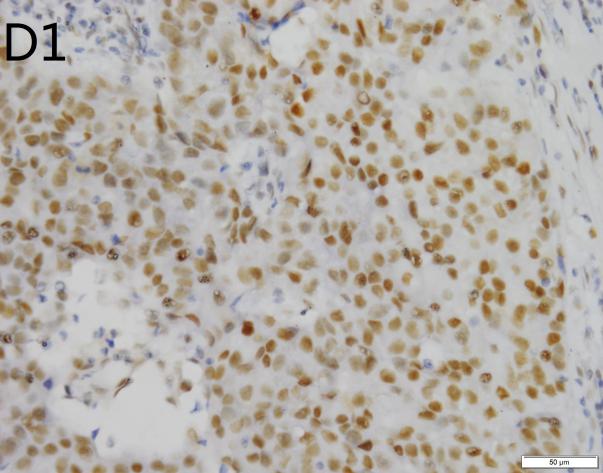

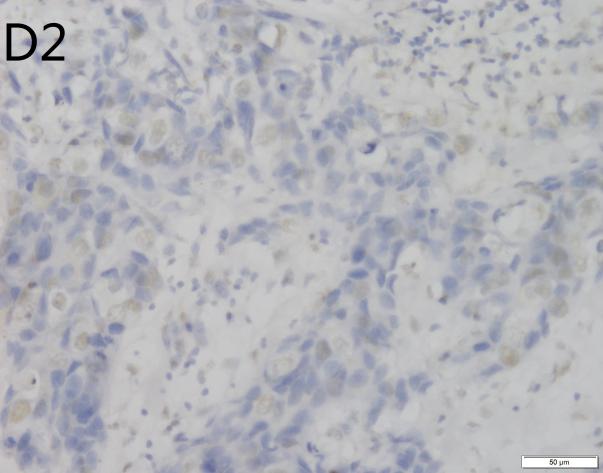

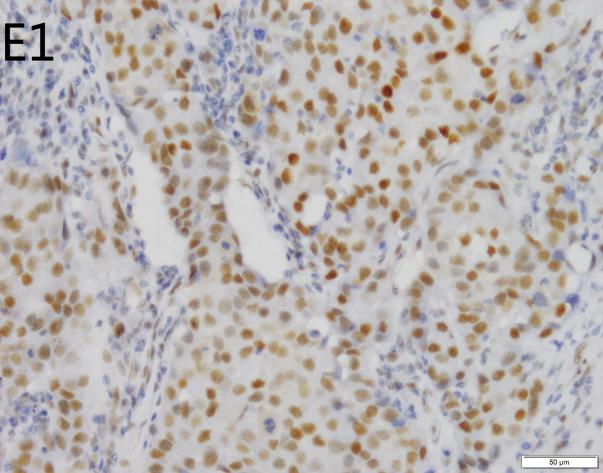

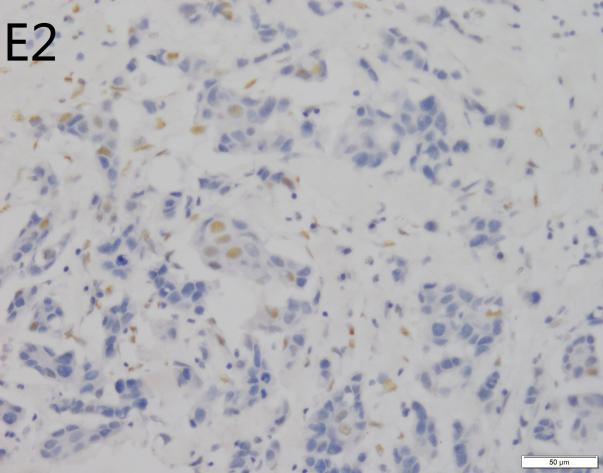

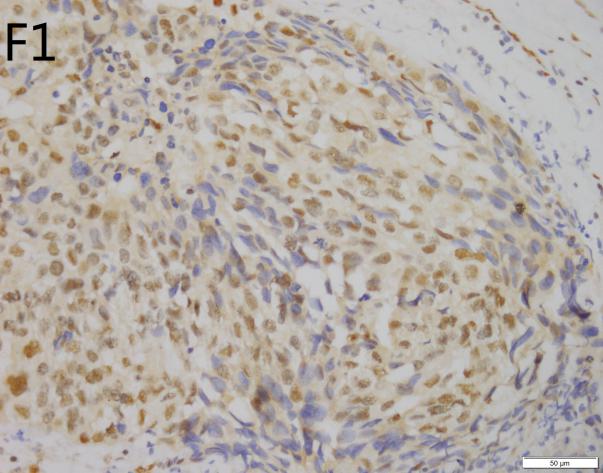

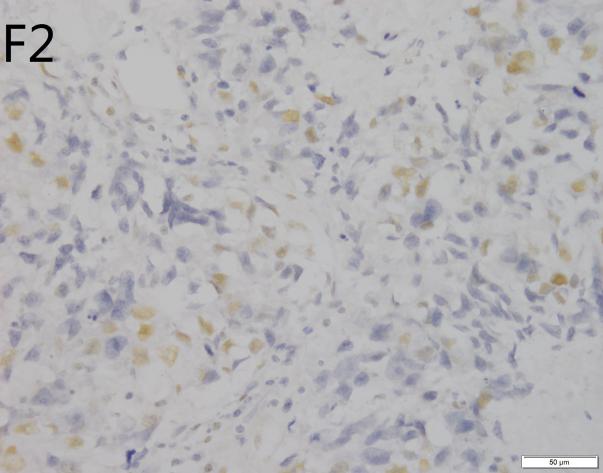

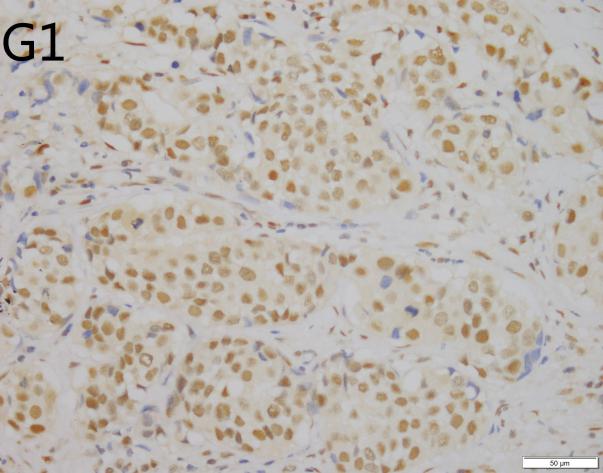

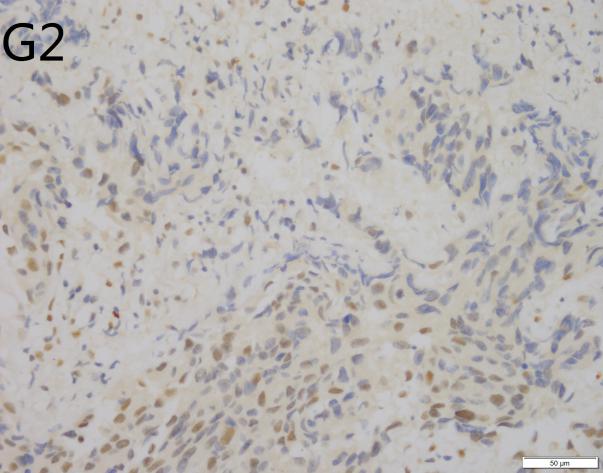

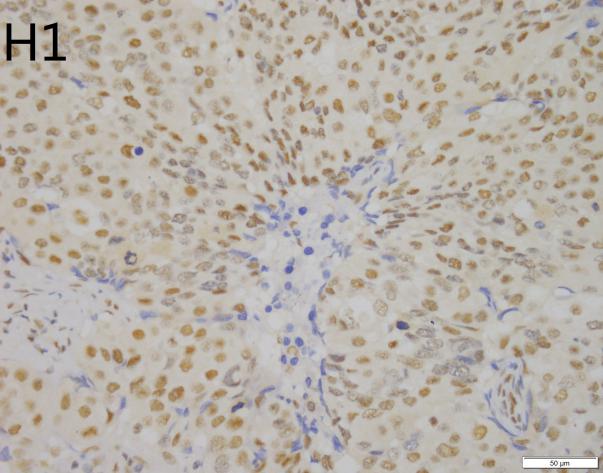

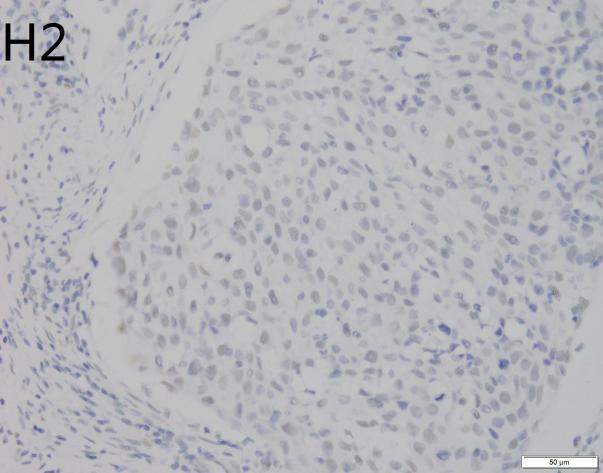

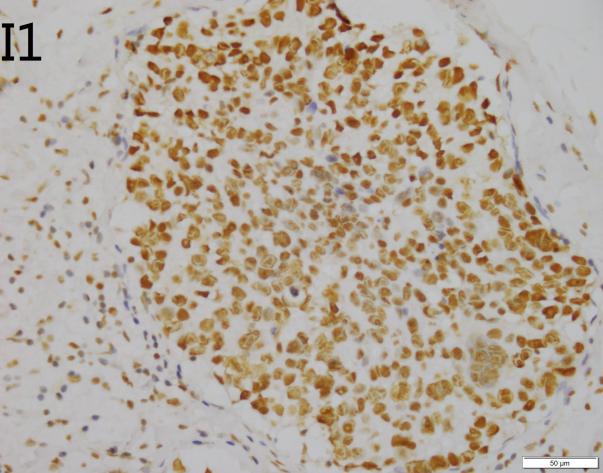

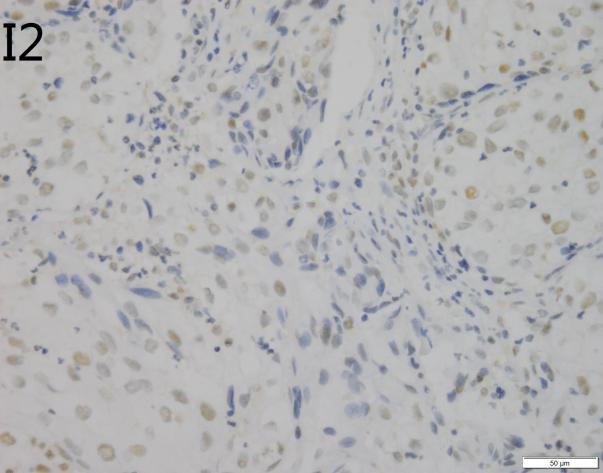


**Supplementary Figure 1**. Strong expression of immunohistochemical positive controls compared to negative controls (A). Immunohistochemistry (IHC) detection of DNA repair genes MSH2 (B), MLH1 (C), PARP1 (D), XRCC1 (E), XRCC4 (F), 53BP1 (G), ERCC1 (H), XPA (I) in paraffin tissues of patients with metastasis breast cancer (1 for the metastasis group, 2 for the control group( metastasis-free group); original magnification × 400).
